# Supplementary material for: Generation of marmoset primordial germ cell–like cells under chemically defined conditions
Source: Life Sci Alliance. 2024 Mar 18;7(6):e202302371. doi: 10.26508/lsa.202302371 (PMC10948935; doi:10.26508/lsa.202302371)
Supplement: Supplementary file 1 [file LSA-2023-02371_TableS1.docx]

**Supplementary Table 1: Compositions of media used to culture cjiPSCs**

| **Name** | **Objective** | **Reference** | **Composition** |
| --- | --- | --- | --- |
| **UPPS** | Reprogramming, maintenance and PGCLC differentiation | Rodríguez-Polo *et al*, 2022) | StemMACS iPS Brew XF supplemented with 1 μM IWR-1 and 0.5 μM CHIR99021 |
| **Modified UPPS** | PGCLC differentiation | Petkov *et al*, 2020 | StemMACS iPS Brew XF supplemented with 1 μM IWR-1, 0.5 μM CHIR, 10 μM Forskolin, 10 ng/ml LIF and 1 ng/ml Activin A |
| **Culture in E8 on feeder cells** | PGCLC differentiation | Commercial culture medium | Essential 8, 5%KSR and 1 μM IWR-1 |
| **TESR E8** | PGCLC differentiation | Commercial culture medium | TESR-E8 |
| **cjPSCM** | PGCLC differentiation | This publication (based on Gao *et al*, 2019) | N2B27 basal media, 1 µM CHIR99021, 0.1 µM A419259, 2.5 µM 2.0 µM IWR-1, 65 µg/ml vitamin C, 10 ng/ml LIF, 1ng/ml TGFβ and 8ng/ml FGF2 |
