# Supplementary material for: Generation of marmoset primordial germ cell–like cells under chemically defined conditions
Source: Life Sci Alliance. 2024 Mar 18;7(6):e202302371. doi: 10.26508/lsa.202302371 (PMC10948935; doi:10.26508/lsa.202302371)
Supplement: Supplementary file 2 [file LSA-2023-02371_TableS2.docx]

**Supplementary Table 2: List of antibodies**

|  | Name | Company | Catalog # | Dilution |
| --- | --- | --- | --- | --- |
| Primary antibodies cjiPSC characterisation | alfa-fetoprotein | Dako | A0008 | 1:100 |
|  | beta-tubulin III | Sigma-Aldrich | T8660 | 1:1000 |
|  | LIN28 | R&D Systems | AF3757 | 1:300 |
|  | NANOG | Cell Signalling | 4903 | 1:400 |
|  | SALL4 | Abcam | ab57577 | 1:200 |
|  | Smooth muscle actin (SMA) | Sigma-Aldrich | A2547 | 1:1000 |
|  | TRA-1-60 | eBioscience | 14-8863 | 1:100 |
|  | TRA 1-81 | eBioscience | 14-8883 | 1:100 |
|  | SOX17 | R&D Systems | AF1924 | 1:750 |
| Primary antibodies PGCLC/PGC characterisation | AP2g | Santa Cruz | sc-12762 | 1:50 |
|  | BLIMP1 | Cell Signalling | 9115 | 1:100 |
|  | NANOG | Cell Signalling | 4903 | 1:100 |
|  | NANOG | R&D | AF1997 | 1:100 |
|  | OCT4A | Cell Signalling | 2890 | 1:300 |
|  | SOX2 | Santa Cruz | sc-17320 | 1:500 |
|  | FOXA2 | Cell Signalling | 8186 | 1:500 |
| Secondary antibodies | Alexa555-goat-α-mouse IgG | Thermo Fisher | A21424 | 1:1000 |
|  | Alexa568-donkey-α-mouse IgG | Thermo Fisher | A10037 | 1:400 |
|  | Alexa488-goat-α-mouse IgG | Thermo Fisher | A11029 | 1:1000 |
|  | Alexa488-goat-α-mouse IgG/IgM | Thermo Fisher | A10680 | 1:1000 |
|  | Alexa488-donkey-α-goat IgG | Thermo Fisher | A11055 | 1:1000 |
|  | Alexa488-donkey-α-rabbit IgG | Thermo Fisher | A21206 | 1:1000 |
|  | Alexa-555-donkey-α-rabbit IgG | Thermo Fisher | A31572 | 1:400 |
