# Supplementary material for: Generation of marmoset primordial germ cell–like cells under chemically defined conditions
Source: Life Sci Alliance. 2024 Mar 18;7(6):e202302371. doi: 10.26508/lsa.202302371 (PMC10948935; doi:10.26508/lsa.202302371)
Supplement: Supplementary file 3 [file LSA-2023-02371_TableS3.docx]

**Supplementary Table 3: Primers**

| **Name** | **Sequence (5’-3’)** |
| --- | --- |
| **WPRE_fw** | GCTATTGCTTCCCGTATGGC |
| **WPRE_rev** | CAAAGGGAGATCCGACTCGT |
| **OriP1_fw** | GGTTCACTACCCTCGTGGAAT |
| **OriP1_rev** | CGGGGCAGTGCATGTAAT |
| **β-actin_fw** | GACCTGACTGACTACCTCATG |
| **β-actin_rev** | GGTAGTTTCGTGGATGCCACA |
| **OCT4_RT-PCR_fw** | GATCGGATCCTTGGGGCGCCTTCCTTC |
| **OCT4_RT-PCR_rev** | CAGGGTGATCCTCTTCTGCTTC |
| **KLF4_RT-PCR_fw** | GGAAGACGATCTTGGCCCCG |
| **KLF4_RT-PCR_rev** | TACTCTAGACAGTGTGGGTCATATCCACTG |
| **c-MYC_RT-PCR_fw** | TAAGAATGCGGCCGCACTGGATTTTTTTCGGGCAGTGG |
| **c-MYC_RT-PCR_rev** | CCTGGATGATGATGTTTTTGATG |
| **β-actin_RT-PCR_fw** | GGTAGTTTCGTGGATGCCACA |
| **β-actin_RT-PCR_rev** | GACCTGACTGACTACCTCATG |
